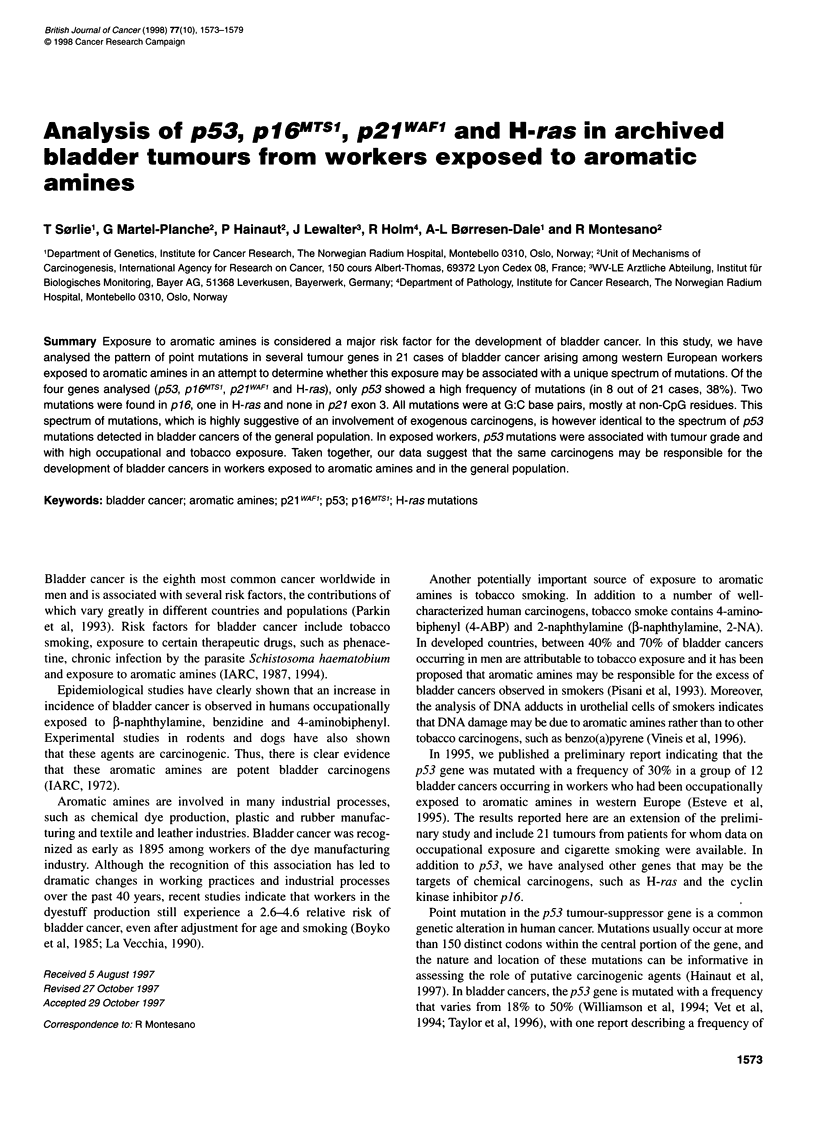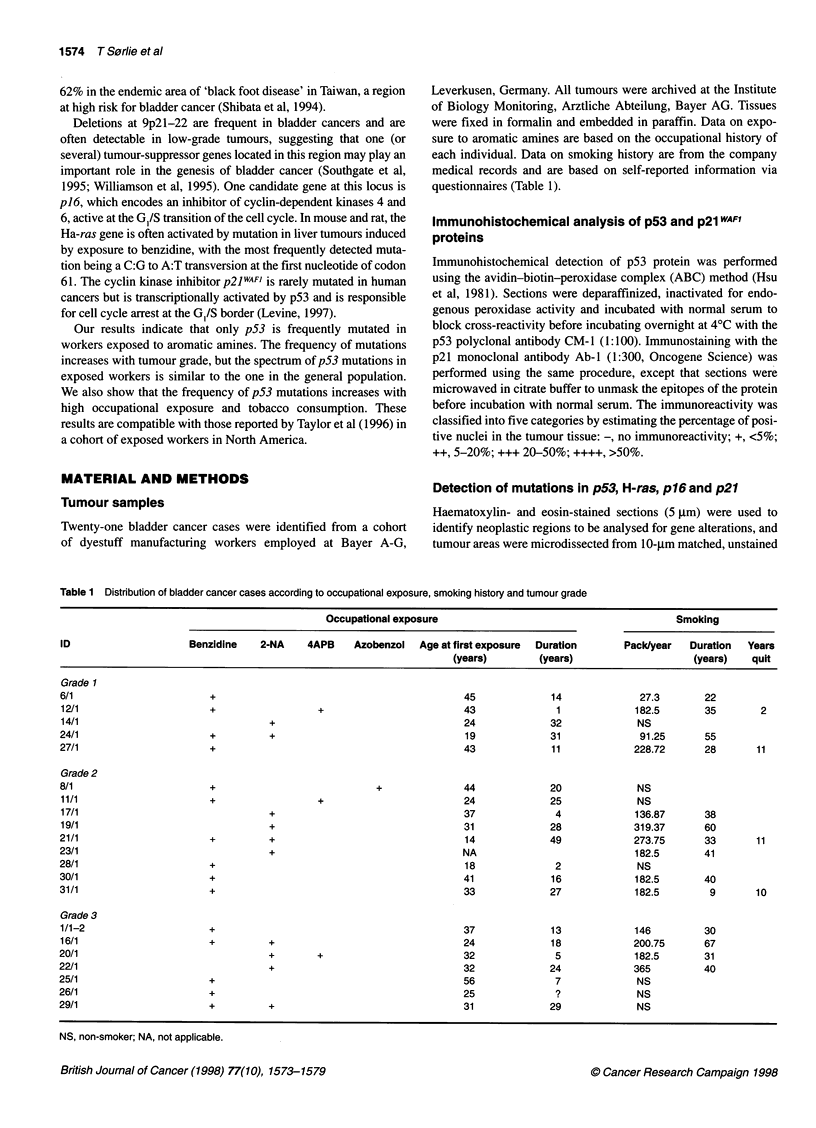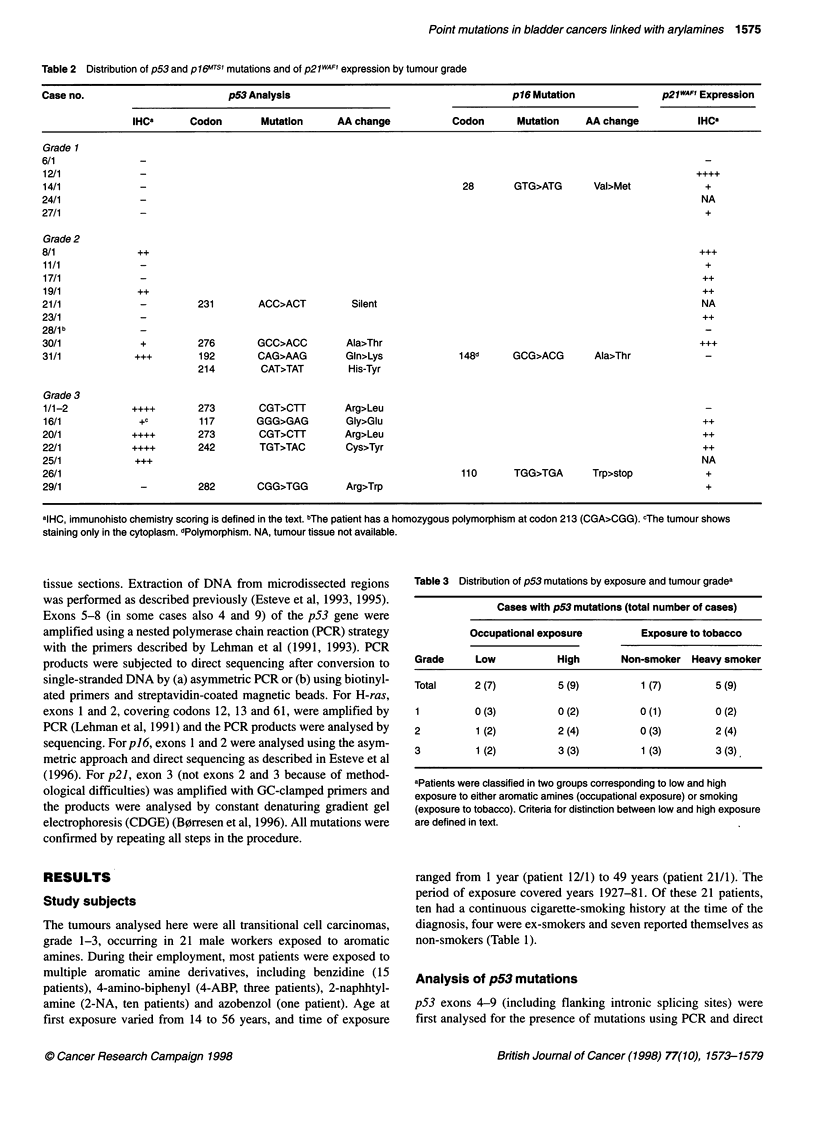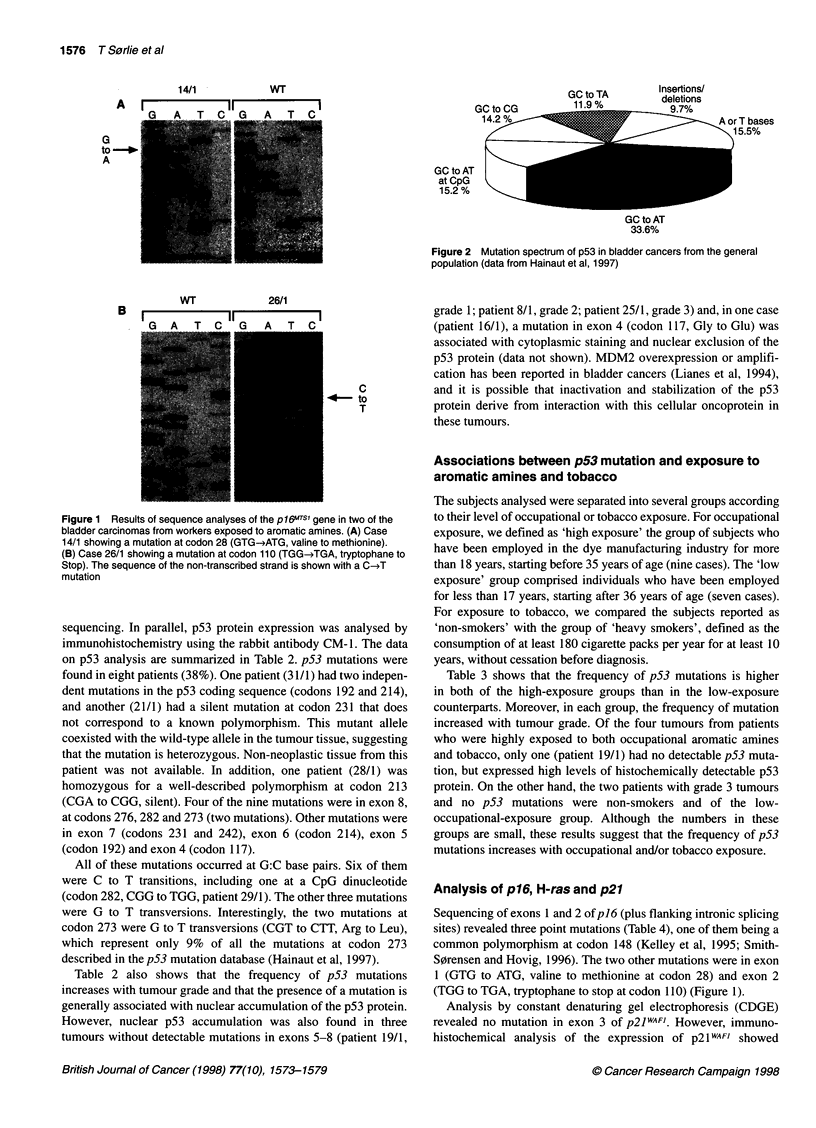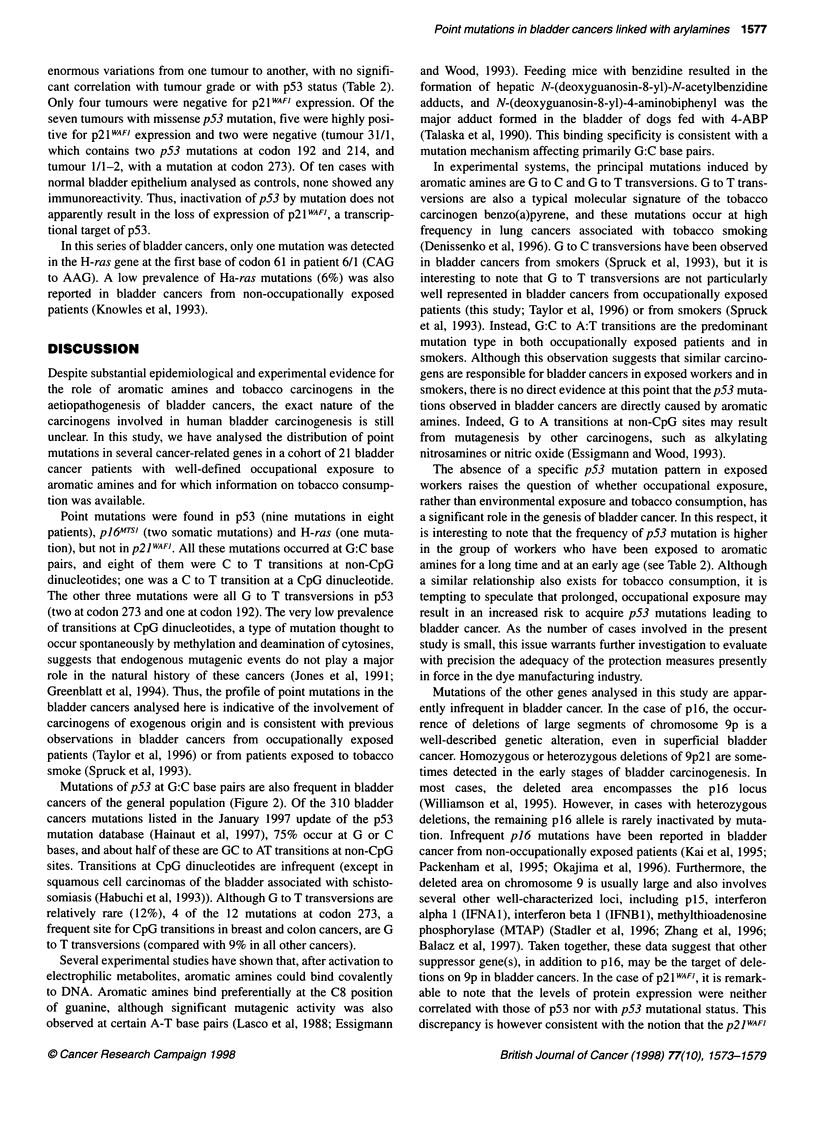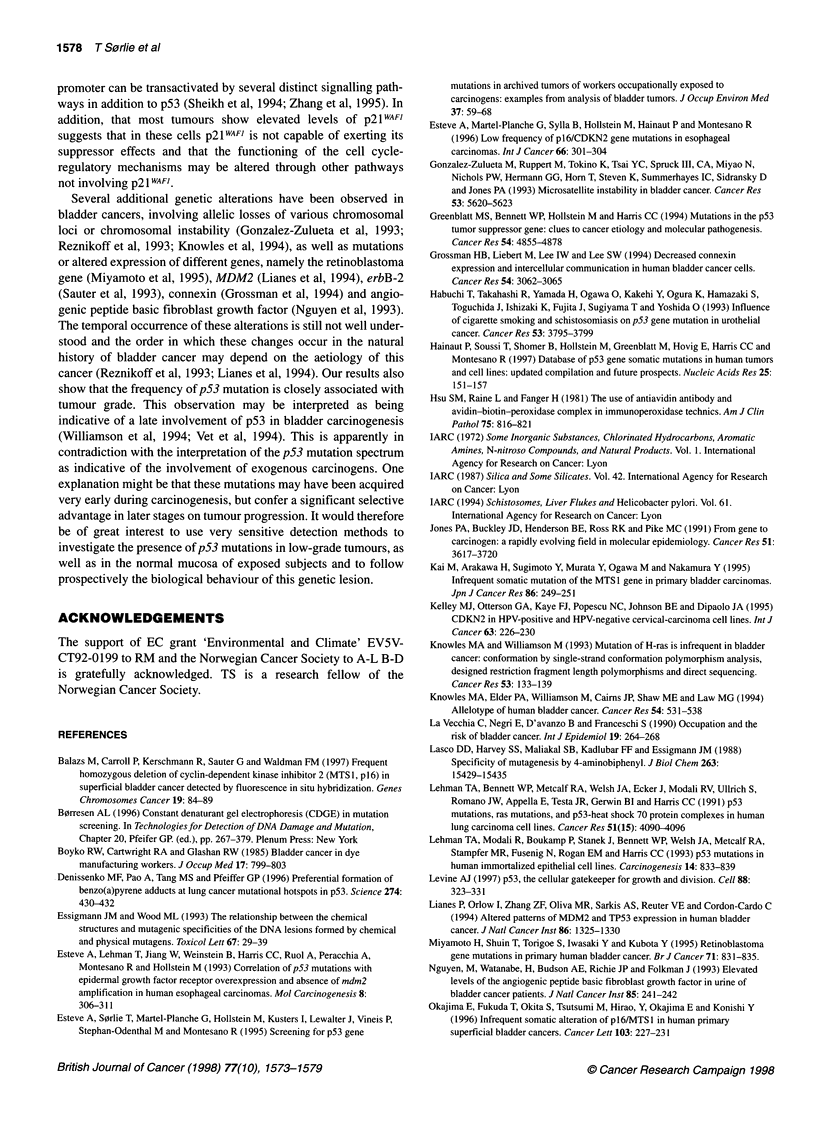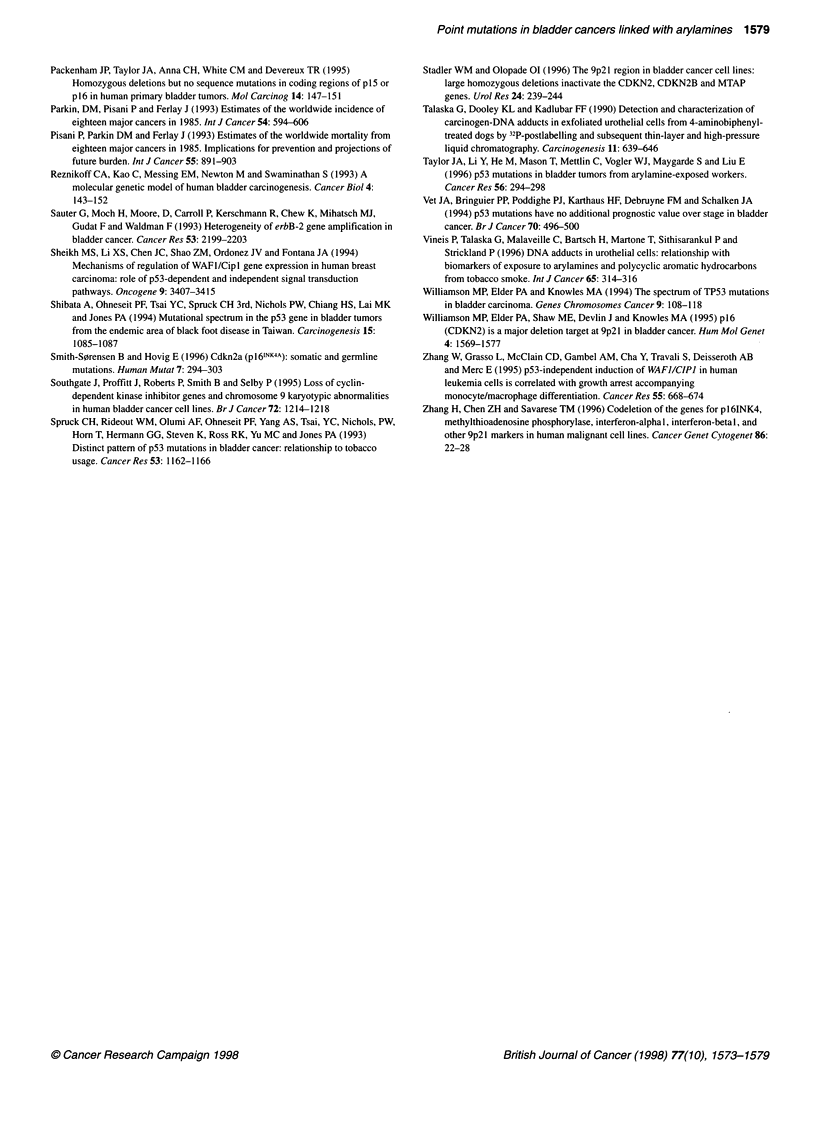# Analysis of p53, p16MTS, p21WAF1 and H-ras in archived bladder tumours from workers exposed to aromatic amines.

**DOI:** 10.1038/bjc.1998.259

**Published:** 1998-05

**Authors:** T. SÃ¸rlie, G. Martel-Planche, P. Hainaut, J. Lewalter, R. Holm, A. L. BÃ¸rresen-Dale, R. Montesano

**Affiliations:** Department of Genetics, Institute for Cancer Research, The Norwegian Radium Hospital, Oslo.

## Abstract

**Images:**


British Journal of Cancer (1998) 77(10), 1573–1579
© 1998 Cancer Research Campaign

### Analysis of *p53*, *p16*$^{MTS1}$, *p21*$^{WAF1}$ and H-*ras* in archived bladder tumours from workers exposed to aromatic amines

T Sørlie[1], G Martel-Planche[2], P Hainaut[2], J Lewalter[3], R Holm[4], A-L Børresen-Dale[1] and R Montesano[2]

[1]Department of Genetics, Institute for Cancer Research, The Norwegian Radium Hospital, Montebello 0310, Oslo, Norway; [2]Unit of Mechanisms of Carcinogenesis, International Agency for Research on Cancer, 150 cours Albert-Thomas, 69372 Lyon Cedex 08, France; [3]WV-LE Arztliche Abteilung, Institut für Biologisches Monitoring, Bayer AG, 51368 Leverkusen, Bayerwerk, Germany; [4]Department of Pathology, Institute for Cancer Research, The Norwegian Radium Hospital, Montebello 0310, Oslo, Norway

**Summary** Exposure to aromatic amines is considered a major risk factor for the development of bladder cancer. In this study, we have analysed the pattern of point mutations in several tumour genes in 21 cases of bladder cancer arising among western European workers exposed to aromatic amines in an attempt to determine whether this exposure may be associated with a unique spectrum of mutations. Of the four genes analysed (*p53*, *p16*$^{MTS1}$, *p21*$^{WAF1}$ and H-*ras*), only *p53* showed a high frequency of mutations (in 8 out of 21 cases, 38%). Two mutations were found in *p16*, one in H-*ras* and none in *p21* exon 3. All mutations were at G:C base pairs, mostly at non-CpG residues. This spectrum of mutations, which is highly suggestive of an involvement of exogenous carcinogens, is however identical to the spectrum of *p53* mutations detected in bladder cancers of the general population. In exposed workers, *p53* mutations were associated with tumour grade and with high occupational and tobacco exposure. Taken together, our data suggest that the same carcinogens may be responsible for the development of bladder cancers in workers exposed to aromatic amines and in the general population.

**Keywords:** bladder cancer; aromatic amines; p21$^{WAF1}$; p53; p16$^{MTS1}$; H-*ras* mutations

Bladder cancer is the eighth most common cancer worldwide in men and is associated with several risk factors, the contributions of which vary greatly in different countries and populations (Parkin et al, 1993). Risk factors for bladder cancer include tobacco smoking, exposure to certain therapeutic drugs, such as phenacetine, chronic infection by the parasite *Schistosoma haematobium* and exposure to aromatic amines (IARC, 1987, 1994).

Epidemiological studies have clearly shown that an increase in incidence of bladder cancer is observed in humans occupationally exposed to β-naphthylamine, benzidine and 4-aminobiphenyl. Experimental studies in rodents and dogs have also shown that these agents are carcinogenic. Thus, there is clear evidence that these aromatic amines are potent bladder carcinogens (IARC, 1972).

Aromatic amines are involved in many industrial processes, such as chemical dye production, plastic and rubber manufacturing and textile and leather industries. Bladder cancer was recognized as early as 1895 among workers of the dye manufacturing industry. Although the recognition of this association has led to dramatic changes in working practices and industrial processes over the past 40 years, recent studies indicate that workers in the dyestuff production still experience a 2.6–4.6 relative risk of bladder cancer, even after adjustment for age and smoking (Boyko et al, 1985; La Vecchia, 1990).

*Received 5 August 1997*
*Revised 27 October 1997*
*Accepted 29 October 1997*

*Correspondence to:* R Montesano

Another potentially important source of exposure to aromatic amines is tobacco smoking. In addition to a number of well-characterized human carcinogens, tobacco smoke contains 4-aminobiphenyl (4-ABP) and 2-naphthylamine (β-naphthylamine, 2-NA). In developed countries, between 40% and 70% of bladder cancers occurring in men are attributable to tobacco exposure and it has been proposed that aromatic amines may be responsible for the excess of bladder cancers observed in smokers (Pisani et al, 1993). Moreover, the analysis of DNA adducts in urothelial cells of smokers indicates that DNA damage may be due to aromatic amines rather than to other tobacco carcinogens, such as benzo(a)pyrene (Vineis et al, 1996).

In 1995, we published a preliminary report indicating that the *p53* gene was mutated with a frequency of 30% in a group of 12 bladder cancers occurring in workers who had been occupationally exposed to aromatic amines in western Europe (Esteve et al, 1995). The results reported here are an extension of the preliminary study and include 21 tumours from patients for whom data on occupational exposure and cigarette smoking were available. In addition to *p53*, we have analysed other genes that may be the targets of chemical carcinogens, such as H-*ras* and the cyclin kinase inhibitor *p16*.

Point mutation in the *p53* tumour-suppressor gene is a common genetic alteration in human cancer. Mutations usually occur at more than 150 distinct codons within the central portion of the gene, and the nature and location of these mutations can be informative in assessing the role of putative carcinogenic agents (Hainaut et al, 1997). In bladder cancers, the *p53* gene is mutated with a frequency that varies from 18% to 50% (Williamson et al, 1994; Vet et al, 1994; Taylor et al, 1996), with one report describing a frequency of

62% in the endemic area of 'black foot disease' in Taiwan, a region at high risk for bladder cancer (Shibata et al, 1994).

Deletions at 9p21–22 are frequent in bladder cancers and are often detectable in low-grade tumours, suggesting that one (or several) tumour-suppressor genes located in this region may play an important role in the genesis of bladder cancer (Southgate et al, 1995; Williamson et al, 1995). One candidate gene at this locus is *p16*, which encodes an inhibitor of cyclin-dependent kinases 4 and 6, active at the $G_1/S$ transition of the cell cycle. In mouse and rat, the Ha-*ras* gene is often activated by mutation in liver tumours induced by exposure to benzidine, with the most frequently detected mutation being a C:G to A:T transversion at the first nucleotide of codon 61. The cyclin kinase inhibitor *p21*$^{WAF1}$ is rarely mutated in human cancers but is transcriptionally activated by p53 and is responsible for cell cycle arrest at the $G_1/S$ border (Levine, 1997).

Our results indicate that only *p53* is frequently mutated in workers exposed to aromatic amines. The frequency of mutations increases with tumour grade, but the spectrum of *p53* mutations in exposed workers is similar to the one in the general population. We also show that the frequency of *p53* mutations increases with high occupational exposure and tobacco consumption. These results are compatible with those reported by Taylor et al (1996) in a cohort of exposed workers in North America.

### MATERIAL AND METHODS

### Tumour samples

Twenty-one bladder cancer cases were identified from a cohort of dyestuff manufacturing workers employed at Bayer A-G, Leverkusen, Germany. All tumours were archived at the Institute of Biology Monitoring, Arztliche Abteilung, Bayer AG. Tissues were fixed in formalin and embedded in paraffin. Data on exposure to aromatic amines are based on the occupational history of each individual. Data on smoking history are from the company medical records and are based on self-reported information via questionnaires (Table 1).

### Immunohistochemical analysis of p53 and p21$^{WAF1}$ proteins

Immunohistochemical detection of p53 protein was performed using the avidin–biotin–peroxidase complex (ABC) method (Hsu et al, 1981). Sections were deparaffinized, inactivated for endogenous peroxidase activity and incubated with normal serum to block cross-reactivity before incubating overnight at 4°C with the p53 polyclonal antibody CM-1 (1:100). Immunostaining with the p21 monoclonal antibody Ab-1 (1:300, Oncogene Science) was performed using the same procedure, except that sections were microwaved in citrate buffer to unmask the epitopes of the protein before incubation with normal serum. The immunoreactivity was classified into five categories by estimating the percentage of positive nuclei in the tumour tissue: –, no immunoreactivity; +, <5%; ++, 5–20%; +++ 20–50%; ++++, >50%.

### Detection of mutations in *p53*, H-*ras*, *p16* and *p21*

Haematoxylin- and eosin-stained sections (5 µm) were used to identify neoplastic regions to be analysed for gene alterations, and tumour areas were microdissected from 10-µm matched, unstained

**Table 1** Distribution of bladder cancer cases according to occupational exposure, smoking history and tumour grade

| ID | Occupational exposure | | | | | | Smoking | | |
|---|---|---|---|---|---|---|---|---|---|
| | Benzidine | 2-NA | 4APB | Azobenzol | Age at first exposure (years) | Duration (years) | Pack/year | Duration (years) | Years quit |
| *Grade 1* | | | | | | | | | |
| 6/1 | + | | | | 45 | 14 | 27.3 | 22 | |
| 12/1 | + | | + | | 43 | 1 | 182.5 | 35 | 2 |
| 14/1 | | + | | | 24 | 32 | NS | | |
| 24/1 | + | + | | | 19 | 31 | 91.25 | 55 | |
| 27/1 | + | | | | 43 | 11 | 228.72 | 28 | 11 |
| *Grade 2* | | | | | | | | | |
| 8/1 | + | | | + | 44 | 20 | NS | | |
| 11/1 | + | | + | | 24 | 25 | NS | | |
| 17/1 | | + | | | 37 | 4 | 136.87 | 38 | |
| 19/1 | | + | | | 31 | 28 | 319.37 | 60 | |
| 21/1 | + | + | | | 14 | 49 | 273.75 | 33 | 11 |
| 23/1 | | + | | | NA | | 182.5 | 41 | |
| 28/1 | + | | | | 18 | 2 | NS | | |
| 30/1 | + | | | | 41 | 16 | 182.5 | 40 | |
| 31/1 | + | | | | 33 | 27 | 182.5 | 9 | 10 |
| *Grade 3* | | | | | | | | | |
| 1/1–2 | + | | | | 37 | 13 | 146 | 30 | |
| 16/1 | + | + | | | 24 | 18 | 200.75 | 67 | |
| 20/1 | | + | + | | 32 | 5 | 182.5 | 31 | |
| 22/1 | | + | | | 32 | 24 | 365 | 40 | |
| 25/1 | + | | | | 56 | 7 | NS | | |
| 26/1 | + | | | | 25 | ? | NS | | |
| 29/1 | + | + | | | 31 | 29 | NS | | |

NS, non-smoker; NA, not applicable.

© Cancer Research Campaign 1998

**Table 2** Distribution of *p53* and $p16^{MTS1}$ mutations and of $p21^{WAF1}$ expression by tumour grade

| Case no. | *p53* Analysis | | | | *p16* Mutation | | | $p21^{WAF1}$ Expression |
|---|---|---|---|---|---|---|---|---|
| | IHC[a] | Codon | Mutation | AA change | Codon | Mutation | AA change | IHC[a] |
| *Grade 1* | | | | | | | | |
| 6/1 | – | | | | | | | – |
| 12/1 | – | | | | | | | ++++ |
| 14/1 | – | | | | 28 | GTG>ATG | Val>Met | + |
| 24/1 | – | | | | | | | NA |
| 27/1 | – | | | | | | | + |
| *Grade 2* | | | | | | | | |
| 8/1 | ++ | | | | | | | +++ |
| 11/1 | – | | | | | | | + |
| 17/1 | – | | | | | | | ++ |
| 19/1 | ++ | | | | | | | ++ |
| 21/1 | – | 231 | ACC>ACT | Silent | | | | NA |
| 23/1 | – | | | | | | | ++ |
| 28/1[b] | – | | | | | | | – |
| 30/1 | + | 276 | GCC>ACC | Ala>Thr | | | | +++ |
| 31/1 | +++ | 192 | CAG>AAG | Gln>Lys | 148[d] | GCG>ACG | Ala>Thr | – |
| | | 214 | CAT>TAT | His-Tyr | | | | |
| *Grade 3* | | | | | | | | |
| 1/1–2 | ++++ | 273 | CGT>CTT | Arg>Leu | | | | – |
| 16/1 | +[c] | 117 | GGG>GAG | Gly>Glu | | | | ++ |
| 20/1 | ++++ | 273 | CGT>CTT | Arg>Leu | | | | ++ |
| 22/1 | ++++ | 242 | TGT>TAC | Cys>Tyr | | | | ++ |
| 25/1 | +++ | | | | | | | NA |
| 26/1 | | | | | 110 | TGG>TGA | Trp>stop | + |
| 29/1 | – | 282 | CGG>TGG | Arg>Trp | | | | + |

[a]IHC, immunohisto chemistry scoring is defined in the text. [b]The patient has a homozygous polymorphism at codon 213 (CGA>CGG). [c]The tumour shows staining only in the cytoplasm. [d]Polymorphism. NA, tumour tissue not available.

tissue sections. Extraction of DNA from microdissected regions was performed as described previously (Esteve et al, 1993, 1995). Exons 5–8 (in some cases also 4 and 9) of the *p53* gene were amplified using a nested polymerase chain reaction (PCR) strategy with the primers described by Lehman et al (1991, 1993). PCR products were subjected to direct sequencing after conversion to single-stranded DNA by (a) asymmetric PCR or (b) using biotinylated primers and streptavidin-coated magnetic beads. For H-*ras*, exons 1 and 2, covering codons 12, 13 and 61, were amplified by PCR (Lehman et al, 1991) and the PCR products were analysed by sequencing. For *p16*, exons 1 and 2 were analysed using the asymmetric approach and direct sequencing as described in Esteve et al (1996). For *p21*, exon 3 (not exons 2 and 3 because of methodological difficulties) was amplified with GC-clamped primers and the products were analysed by constant denaturing gradient gel electrophoresis (CDGE) (Børresen et al, 1996). All mutations were confirmed by repeating all steps in the procedure.

**Table 3** Distribution of *p53* mutations by exposure and tumour grade[a]

| Grade | Cases with *p53* mutations (total number of cases) | | | |
|---|---|---|---|---|
| | Occupational exposure | | Exposure to tobacco | |
| | Low | High | Non-smoker | Heavy smoker |
| Total | 2 (7) | 5 (9) | 1 (7) | 5 (9) |
| 1 | 0 (3) | 0 (2) | 0 (1) | 0 (2) |
| 2 | 1 (2) | 2 (4) | 0 (3) | 2 (4) |
| 3 | 1 (2) | 3 (3) | 1 (3) | 3 (3) |

[a]Patients were classified in two groups corresponding to low and high exposure to either aromatic amines (occupational exposure) or smoking (exposure to tobacco). Criteria for distinction between low and high exposure are defined in text.

### RESULTS

### Study subjects

The tumours analysed here were all transitional cell carcinomas, grade 1–3, occurring in 21 male workers exposed to aromatic amines. During their employment, most patients were exposed to multiple aromatic amine derivatives, including benzidine (15 patients), 4-amino-biphenyl (4-ABP, three patients), 2-naphhtylamine (2-NA, ten patients) and azobenzol (one patient). Age at first exposure varied from 14 to 56 years, and time of exposure ranged from 1 year (patient 12/1) to 49 years (patient 21/1). The period of exposure covered years 1927–81. Of these 21 patients, ten had a continuous cigarette-smoking history at the time of the diagnosis, four were ex-smokers and seven reported themselves as non-smokers (Table 1).

### Analysis of *p53* mutations

*p53* exons 4–9 (including flanking intronic splicing sites) were first analysed for the presence of mutations using PCR and direct

© Cancer Research Campaign 1998

Figure 1 Results of sequence analyses of the $p16^{MTS1}$ gene in two of the bladder carcinomas from workers exposed to aromatic amines. (**A**) Case 14/1 showing a mutation at codon 28 (GTG→ATG, valine to methionine). (**B**) Case 26/1 showing a mutation at codon 110 (TGG→TGA, tryptophane to Stop). The sequence of the non-transcribed strand is shown with a C→T mutation

**Figure 2** Mutation spectrum of p53 in bladder cancers from the general population (data from Hainaut et al, 1997)

sequencing. In parallel, p53 protein expression was analysed by immunohistochemistry using the rabbit antibody CM-1. The data on p53 analysis are summarized in Table 2. *p53* mutations were found in eight patients (38%). One patient (31/1) had two independent mutations in the p53 coding sequence (codons 192 and 214), and another (21/1) had a silent mutation at codon 231 that does not correspond to a known polymorphism. This mutant allele coexisted with the wild-type allele in the tumour tissue, suggesting that the mutation is heterozygous. Non-neoplastic tissue from this patient was not available. In addition, one patient (28/1) was homozygous for a well-described polymorphism at codon 213 (CGA to CGG, silent). Four of the nine mutations were in exon 8, at codons 276, 282 and 273 (two mutations). Other mutations were in exon 7 (codons 231 and 242), exon 6 (codon 214), exon 5 (codon 192) and exon 4 (codon 117).

All of these mutations occurred at G:C base pairs. Six of them were C to T transitions, including one at a CpG dinucleotide (codon 282, CGG to TGG, patient 29/1). The other three mutations were G to T transversions. Interestingly, the two mutations at codon 273 were G to T transversions (CGT to CTT, Arg to Leu), which represent only 9% of all the mutations at codon 273 described in the *p53* mutation database (Hainaut et al, 1997).

Table 2 also shows that the frequency of *p53* mutations increases with tumour grade and that the presence of a mutation is generally associated with nuclear accumulation of the p53 protein. However, nuclear p53 accumulation was also found in three tumours without detectable mutations in exons 5–8 (patient 19/1, grade 1; patient 8/1, grade 2; patient 25/1, grade 3) and, in one case (patient 16/1), a mutation in exon 4 (codon 117, Gly to Glu) was associated with cytoplasmic staining and nuclear exclusion of the p53 protein (data not shown). MDM2 overexpression or amplification has been reported in bladder cancers (Lianes et al, 1994), and it is possible that inactivation and stabilization of the p53 protein derive from interaction with this cellular oncoprotein in these tumours.

### Associations between *p53* mutation and exposure to aromatic amines and tobacco

The subjects analysed were separated into several groups according to their level of occupational or tobacco exposure. For occupational exposure, we defined as 'high exposure' the group of subjects who have been employed in the dye manufacturing industry for more than 18 years, starting before 35 years of age (nine cases). The 'low exposure' group comprised individuals who have been employed for less than 17 years, starting after 36 years of age (seven cases). For exposure to tobacco, we compared the subjects reported as 'non-smokers' with the group of 'heavy smokers', defined as the consumption of at least 180 cigarette packs per year for at least 10 years, without cessation before diagnosis.

Table 3 shows that the frequency of *p53* mutations is higher in both of the high-exposure groups than in the low-exposure counterparts. Moreover, in each group, the frequency of mutation increased with tumour grade. Of the four tumours from patients who were highly exposed to both occupational aromatic amines and tobacco, only one (patient 19/1) had no detectable *p53* mutation, but expressed high levels of histochemically detectable p53 protein. On the other hand, the two patients with grade 3 tumours and no *p53* mutations were non-smokers and of the low-occupational-exposure group. Although the numbers in these groups are small, these results suggest that the frequency of *p53* mutations increases with occupational and/or tobacco exposure.

### Analysis of *p16*, H-*ras* and *p21*

Sequencing of exons 1 and 2 of *p16* (plus flanking intronic splicing sites) revealed three point mutations (Table 4), one of them being a common polymorphism at codon 148 (Kelley et al, 1995; Smith-Sørensen and Hovig, 1996). The two other mutations were in exon 1 (GTG to ATG, valine to methionine at codon 28) and exon 2 (TGG to TGA, tryptophane to stop at codon 110) (Figure 1).

Analysis by constant denaturing gel electrophoresis (CDGE) revealed no mutation in exon 3 of $p21^{WAF1}$. However, immunohistochemical analysis of the expression of $p21^{WAF1}$ showed

 © Cancer Research Campaign 1998

enormous variations from one tumour to another, with no significant correlation with tumour grade or with p53 status (Table 2). Only four tumours were negative for $p21^{WAF1}$ expression. Of the seven tumours with missense *p53* mutation, five were highly positive for $p21^{WAF1}$ expression and two were negative (tumour 31/1, which contains two *p53* mutations at codon 192 and 214, and tumour 1/1–2, with a mutation at codon 273). Of ten cases with normal bladder epithelium analysed as controls, none showed any immunoreactivity. Thus, inactivation of *p53* by mutation does not apparently result in the loss of expression of $p21^{WAF1}$, a transcriptional target of p53.

In this series of bladder cancers, only one mutation was detected in the H-*ras* gene at the first base of codon 61 in patient 6/1 (CAG to AAG). A low prevalence of Ha-*ras* mutations (6%) was also reported in bladder cancers from non-occupationally exposed patients (Knowles et al, 1993).

### DISCUSSION

Despite substantial epidemiological and experimental evidence for the role of aromatic amines and tobacco carcinogens in the aetiopathogenesis of bladder cancers, the exact nature of the carcinogens involved in human bladder carcinogenesis is still unclear. In this study, we have analysed the distribution of point mutations in several cancer-related genes in a cohort of 21 bladder cancer patients with well-defined occupational exposure to aromatic amines and for which information on tobacco consumption was available.

Point mutations were found in p53 (nine mutations in eight patients), $p16^{MTS1}$ (two somatic mutations) and H-*ras* (one mutation), but not in $p21^{WAF1}$. All these mutations occurred at G:C base pairs, and eight of them were C to T transitions at non-CpG dinucleotides; one was a C to T transition at a CpG dinucleotide. The other three mutations were all G to T transversions in p53 (two at codon 273 and one at codon 192). The very low prevalence of transitions at CpG dinucleotides, a type of mutation thought to occur spontaneously by methylation and deamination of cytosines, suggests that endogenous mutagenic events do not play a major role in the natural history of these cancers (Jones et al, 1991; Greenblatt et al, 1994). Thus, the profile of point mutations in the bladder cancers analysed here is indicative of the involvement of carcinogens of exogenous origin and is consistent with previous observations in bladder cancers from occupationally exposed patients (Taylor et al, 1996) or from patients exposed to tobacco smoke (Spruck et al, 1993).

Mutations of *p53* at G:C base pairs are also frequent in bladder cancers of the general population (Figure 2). Of the 310 bladder cancers mutations listed in the January 1997 update of the p53 mutation database (Hainaut et al, 1997), 75% occur at G or C bases, and about half of these are GC to AT transitions at non-CpG sites. Transitions at CpG dinucleotides are infrequent (except in squamous cell carcinomas of the bladder associated with schistosomiasis (Habuchi et al, 1993)). Although G to T transversions are relatively rare (12%), 4 of the 12 mutations at codon 273, a frequent site for CpG transitions in breast and colon cancers, are G to T transversions (compared with 9% in all other cancers).

Several experimental studies have shown that, after activation to electrophilic metabolites, aromatic amines could bind covalently to DNA. Aromatic amines bind preferentially at the C8 position of guanine, although significant mutagenic activity was also observed at certain A-T base pairs (Lasco et al, 1988; Essigmann and Wood, 1993). Feeding mice with benzidine resulted in the formation of hepatic *N*-(deoxyguanosin-8-yl)-*N*-acetylbenzidine adducts, and *N*-(deoxyguanosin-8-yl)-4-aminobiphenyl was the major adduct formed in the bladder of dogs fed with 4-ABP (Talaska et al, 1990). This binding specificity is consistent with a mutation mechanism affecting primarily G:C base pairs.

In experimental systems, the principal mutations induced by aromatic amines are G to C and G to T transversions. G to T transversions are also a typical molecular signature of the tobacco carcinogen benzo(a)pyrene, and these mutations occur at high frequency in lung cancers associated with tobacco smoking (Denissenko et al, 1996). G to C transversions have been observed in bladder cancers from smokers (Spruck et al, 1993), but it is interesting to note that G to T transversions are not particularly well represented in bladder cancers from occupationally exposed patients (this study; Taylor et al, 1996) or from smokers (Spruck et al, 1993). Instead, G:C to A:T transitions are the predominant mutation type in both occupationally exposed patients and in smokers. Although this observation suggests that similar carcinogens are responsible for bladder cancers in exposed workers and in smokers, there is no direct evidence at this point that the *p53* mutations observed in bladder cancers are directly caused by aromatic amines. Indeed, G to A transitions at non-CpG sites may result from mutagenesis by other carcinogens, such as alkylating nitrosamines or nitric oxide (Essigmann and Wood, 1993).

The absence of a specific *p53* mutation pattern in exposed workers raises the question of whether occupational exposure, rather than environmental exposure and tobacco consumption, has a significant role in the genesis of bladder cancer. In this respect, it is interesting to note that the frequency of *p53* mutation is higher in the group of workers who have been exposed to aromatic amines for a long time and at an early age (see Table 2). Although a similar relationship also exists for tobacco consumption, it is tempting to speculate that prolonged, occupational exposure may result in an increased risk to acquire *p53* mutations leading to bladder cancer. As the number of cases involved in the present study is small, this issue warrants further investigation to evaluate with precision the adequacy of the protection measures presently in force in the dye manufacturing industry.

Mutations of the other genes analysed in this study are apparently infrequent in bladder cancer. In the case of p16, the occurrence of deletions of large segments of chromosome 9p is a well-described genetic alteration, even in superficial bladder cancer. Homozygous or heterozygous deletions of 9p21 are sometimes detected in the early stages of bladder carcinogenesis. In most cases, the deleted area encompasses the p16 locus (Williamson et al, 1995). However, in cases with heterozygous deletions, the remaining p16 allele is rarely inactivated by mutation. Infrequent *p16* mutations have been reported in bladder cancer from non-occupationally exposed patients (Kai et al, 1995; Packenham et al, 1995; Okajima et al, 1996). Furthermore, the deleted area on chromosome 9 is usually large and also involves several other well-characterized loci, including p15, interferon alpha 1 (IFNA1), interferon beta 1 (IFNB1), methylthioadenosine phosphorylase (MTAP) (Stadler et al, 1996; Zhang et al, 1996; Balacz et al, 1997). Taken together, these data suggest that other suppressor gene(s), in addition to p16, may be the target of deletions on 9p in bladder cancers. In the case of $p21^{WAF1}$, it is remarkable to note that the levels of protein expression were neither correlated with those of p53 nor with *p53* mutational status. This discrepancy is however consistent with the notion that the $p21^{WAF1}$

© Cancer Research Campaign 1998

promoter can be transactivated by several distinct signalling pathways in addition to p53 (Sheikh et al, 1994; Zhang et al, 1995). In addition, that most tumours show elevated levels of p21$^{WAF1}$ suggests that in these cells p21$^{WAF1}$ is not capable of exerting its suppressor effects and that the functioning of the cell cycle-regulatory mechanisms may be altered through other pathways not involving p21$^{WAF1}$.

Several additional genetic alterations have been observed in bladder cancers, involving allelic losses of various chromosomal loci or chromosomal instability (Gonzalez-Zulueta et al, 1993; Reznikoff et al, 1993; Knowles et al, 1994), as well as mutations or altered expression of different genes, namely the retinoblastoma gene (Miyamoto et al, 1995), *MDM2* (Lianes et al, 1994), *erb*B-2 (Sauter et al, 1993), connexin (Grossman et al, 1994) and angiogenic peptide basic fibroblast growth factor (Nguyen et al, 1993). The temporal occurrence of these alterations is still not well understood and the order in which these changes occur in the natural history of bladder cancer may depend on the aetiology of this cancer (Reznikoff et al, 1993; Lianes et al, 1994). Our results also show that the frequency of *p53* mutation is closely associated with tumour grade. This observation may be interpreted as being indicative of a late involvement of p53 in bladder carcinogenesis (Williamson et al, 1994; Vet et al, 1994). This is apparently in contradiction with the interpretation of the *p53* mutation spectrum as indicative of the involvement of exogenous carcinogens. One explanation might be that these mutations may have been acquired very early during carcinogenesis, but confer a significant selective advantage in later stages on tumour progression. It would therefore be of great interest to use very sensitive detection methods to investigate the presence of *p53* mutations in low-grade tumours, as well as in the normal mucosa of exposed subjects and to follow prospectively the biological behaviour of this genetic lesion.

### ACKNOWLEDGEMENTS

The support of EC grant 'Environmental and Climate' EV5V-CT92-0199 to RM and the Norwegian Cancer Society to A-L B-D is gratefully acknowledged. TS is a research fellow of the Norwegian Cancer Society.

### REFERENCES

Balazs M, Carroll P, Kerschmann R, Sauter G and Waldman FM (1997) Frequent homozygous deletion of cyclin-dependent kinase inhibitor 2 (MTS1, p16) in superficial bladder cancer detected by fluorescence in situ hybridization. *Genes Chromosomes Cancer* **19**: 84–89

Børresen AL (1996) Constant denaturant gel electrophoresis (CDGE) in mutation screening. In *Technologies for Detection of DNA Damage and Mutation*, Chapter 20, Pfeifer GP. (ed.), pp. 267–379. Plenum Press: New York

Boyko RW, Cartwright RA and Glashan RW (1985) Bladder cancer in dye manufacturing workers. *J Occup Med* **17**: 799–803

Denissenko MF, Pao A, Tang MS and Pfeiffer GP (1996) Preferential formation of benzo(a)pyrene adducts at lung cancer mutational hotspots in p53. *Science* **274**: 430–432

Essigmann JM and Wood ML (1993) The relationship between the chemical structures and mutagenic specificities of the DNA lesions formed by chemical and physical mutagens. *Toxicol Lett* **67**: 29–39

Esteve A, Lehman T, Jiang W, Weinstein B, Harris CC, Ruol A, Peracchia A, Montesano R and Hollstein M (1993) Correlation of *p53* mutations with epidermal growth factor receptor overexpression and absence of *mdm2* amplification in human esophageal carcinomas. *Mol Carcinogenesis* **8**: 306–311

Esteve A, Sørlie T, Martel-Planche G, Hollstein M, Kusters I, Lewalter J, Vineis P, Stephan-Odenthal M and Montesano R (1995) Screening for p53 gene mutations in archived tumors of workers occupationally exposed to carcinogens: examples from analysis of bladder tumors. *J Occup Environ Med* **37**: 59–68

Esteve A, Martel-Planche G, Sylla B, Hollstein M, Hainaut P and Montesano R (1996) Low frequency of p16/CDKN2 gene mutations in esophageal carcinomas. *Int J Cancer* **66**: 301–304

Gonzalez-Zulueta M, Ruppert M, Tokino K, Tsai YC, Spruck III, CA, Miyao N, Nichols PW, Hermann GG, Horn T, Steven K, Summerhayes IC, Sidransky D and Jones PA (1993) Microsatellite instability in bladder cancer. *Cancer Res* **53**: 5620–5623

Greenblatt MS, Bennett WP, Hollstein M and Harris CC (1994) Mutations in the p53 tumor suppressor gene: clues to cancer etiology and molecular pathogenesis. *Cancer Res* **54**: 4855–4878

Grossman HB, Liebert M, Lee IW and Lee SW (1994) Decreased connexin expression and intercellular communication in human bladder cancer cells. *Cancer Res* **54**: 3062–3065

Habuchi T, Takahashi R, Yamada H, Ogawa O, Kakehi Y, Ogura K, Hamazaki S, Toguchida J, Ishizaki K, Fujita J, Sugiyama T and Yoshida O (1993) Influence of cigarette smoking and schistosomiasis on *p53* gene mutation in urothelial cancer. *Cancer Res* **53**: 3795–3799

Hainaut P, Soussi T, Shomer B, Hollstein M, Greenblatt M, Hovig E, Harris CC and Montesano R (1997) Database of p53 gene somatic mutations in human tumors and cell lines: updated compilation and future prospects. *Nucleic Acids Res* **25**: 151–157

Hsu SM, Raine L and Fanger H (1981) The use of antiavidin antibody and avidin–biotin–peroxidase complex in immunoperoxidase technics. *Am J Clin Pathol* **75**: 816–821

IARC (1972) *Some Inorganic Substances, Chlorinated Hydrocarbons, Aromatic Amines, N-nitroso Compounds, and Natural Products*. Vol. 1. International Agency for Research on Cancer: Lyon

IARC (1987) *Silica and Some Silicates*. Vol. 42. International Agency for Research on Cancer: Lyon

IARC (1994) *Schistosomes, Liver Flukes and* Helicobacter pylori. Vol. 61. International Agency for Research on Cancer: Lyon

Jones PA, Buckley JD, Henderson BE, Ross RK and Pike MC (1991) From gene to carcinogen: a rapidly evolving field in molecular epidemiology. *Cancer Res* **51**: 3617–3720

Kai M, Arakawa H, Sugimoto Y, Murata Y, Ogawa M and Nakamura Y (1995) Infrequent somatic mutation of the MTS1 gene in primary bladder carcinomas. *Jpn J Cancer Res* **86**: 249–251

Kelley MJ, Otterson GA, Kaye FJ, Popescu NC, Johnson BE and Dipaolo JA (1995) CDKN2 in HPV-positive and HPV-negative cervical-carcinoma cell lines. *Int J Cancer* **63**: 226–230

Knowles MA and Williamson M (1993) Mutation of H-ras is infrequent in bladder cancer: conformation by single-strand conformation polymorphism analysis, designed restriction fragment length polymorphisms and direct sequencing. *Cancer Res* **53**: 133–139

Knowles MA, Elder PA, Williamson M, Cairns JP, Shaw ME and Law MG (1994) Allelotype of human bladder cancer. *Cancer Res* **54**: 531–538

La Vecchia C, Negri E, D'avanzo B and Franceschi S (1990) Occupation and the risk of bladder cancer. *Int J Epidemiol* **19**: 264–268

Lasco DD, Harvey SS, Maliakal SB, Kadlubar FF and Essigmann JM (1988) Specificity of mutagenesis by 4-aminobiphenyl. *J Biol Chem* **263**: 15429–15435

Lehman TA, Bennett WP, Metcalf RA, Welsh JA, Ecker J, Modali RV, Ullrich S, Romano JW, Appella E, Testa JR, Gerwin BI and Harris CC (1991) p53 mutations, ras mutations, and p53-heat shock 70 protein complexes in human lung carcinoma cell lines. *Cancer Res* **51(15)**: 4090–4096

Lehman TA, Modali R, Boukamp P, Stanek J, Bennett WP, Welsh JA, Metcalf RA, Stampfer MR, Fusenig N, Rogan EM and Harris CC (1993) p53 mutations in human immortalized epithelial cell lines. *Carcinogenesis* **14**: 833–839

Levine AJ (1997) p53, the cellular gatekeeper for growth and division. *Cell* **88**: 323–331

Lianes P, Orlow I, Zhang ZF, Oliva MR, Sarkis AS, Reuter VE and Cordon-Cardo C (1994) Altered patterns of MDM2 and TP53 expression in human bladder cancer. *J Natl Cancer Inst* **86**: 1325–1330

Miyamoto H, Shuin T, Torigoe S, Iwasaki Y and Kubota Y (1995) Retinoblastoma gene mutations in primary human bladder cancer. *Br J Cancer* **71**: 831–835.

Nguyen, M, Watanabe, H, Budson AE, Richie JP and Folkman J (1993) Elevated levels of the angiogenic peptide basic fibroblast growth factor in urine of bladder cancer patients. *J Natl Cancer Inst* **85**: 241–242

Okajima E, Fukuda T, Okita S, Tsutsumi M, Hirao, Y, Okajima E and Konishi Y (1996) Infrequent somatic alteration of p16/MTS1 in human primary superficial bladder cancers. *Cancer Lett* **103**: 227–231

 © Cancer Research Campaign 1998

Packenham JP, Taylor JA, Anna CH, White CM and Devereux TR (1995) Homozygous deletions but no sequence mutations in coding regions of p15 or p16 in human primary bladder tumors. *Mol Carcinog* **14**: 147–151

Parkin, DM, Pisani P and Ferlay J (1993) Estimates of the worldwide incidence of eighteen major cancers in 1985. *Int J Cancer* **54**: 594–606

Pisani P, Parkin DM and Ferlay J (1993) Estimates of the worldwide mortality from eighteen major cancers in 1985. Implications for prevention and projections of future burden. *Int J Cancer* **55**: 891–903

Reznikoff CA, Kao C, Messing EM, Newton M and Swaminathan S (1993) A molecular genetic model of human bladder carcinogenesis. *Cancer Biol* **4**: 143–152

Sauter G, Moch H, Moore, D, Carroll P, Kerschmann R, Chew K, Mihatsch MJ, Gudat F and Waldman F (1993) Heterogeneity of *erb*B-2 gene amplification in bladder cancer. *Cancer Res* **53**: 2199–2203

Sheikh MS, Li XS, Chen JC, Shao ZM, Ordonez JV and Fontana JA (1994) Mechanisms of regulation of WAF1/Cip1 gene expression in human breast carcinoma: role of p53-dependent and independent signal transduction pathways. *Oncogene* **9**: 3407–3415

Shibata A, Ohneseit PF, Tsai YC, Spruck CH 3rd, Nichols PW, Chiang HS, Lai MK and Jones PA (1994) Mutational spectrum in the p53 gene in bladder tumors from the endemic area of black foot disease in Taiwan. *Carcinogenesis* **15**: 1085–1087

Smith-Sørensen B and Hovig E (1996) Cdkn2a ($p16^{INK4A}$): somatic and germline mutations. *Human Mutat* **7**: 294–303

Southgate J, Proffitt J, Roberts P, Smith B and Selby P (1995) Loss of cyclin-dependent kinase inhibitor genes and chromosome 9 karyotypic abnormalities in human bladder cancer cell lines. *Br J Cancer* **72**: 1214–1218

Spruck CH, Rideout WM, Olumi AF, Ohneseit PF, Yang AS, Tsai, YC, Nichols, PW, Horn T, Hermann GG, Steven K, Ross RK, Yu MC and Jones PA (1993) Distinct pattern of p53 mutations in bladder cancer: relationship to tobacco usage. *Cancer Res* **53**: 1162–1166

Stadler WM and Olopade OI (1996) The 9p21 region in bladder cancer cell lines: large homozygous deletions inactivate the CDKN2, CDKN2B and MTAP genes. *Urol Res* **24**: 239–244

Talaska G, Dooley KL and Kadlubar FF (1990) Detection and characterization of carcinogen-DNA adducts in exfoliated urothelial cells from 4-aminobiphenyl-treated dogs by $^{32}P$-postlabelling and subsequent thin-layer and high-pressure liquid chromatography. *Carcinogenesis* **11**: 639–646

Taylor JA, Li Y, He M, Mason T, Mettlin C, Vogler WJ, Maygarde S and Liu E (1996) p53 mutations in bladder tumors from arylamine-exposed workers. *Cancer Res* **56**: 294–298

Vet JA, Bringuier PP, Poddighe PJ, Karthaus HF, Debruyne FM and Schalken JA (1994) p53 mutations have no additional prognostic value over stage in bladder cancer. *Br J Cancer* **70**: 496–500

Vineis P, Talaska G, Malaveille C, Bartsch H, Martone T, Sithisarankul P and Strickland P (1996) DNA adducts in urothelial cells: relationship with biomarkers of exposure to arylamines and polycyclic aromatic hydrocarbons from tobacco smoke. *Int J Cancer* **65**: 314–316

Williamson MP, Elder PA and Knowles MA (1994) The spectrum of TP53 mutations in bladder carcinoma. *Genes Chromosomes Cancer* **9**: 108–118

Williamson MP, Elder PA, Shaw ME, Devlin J and Knowles MA (1995) p16 (CDKN2) is a major deletion target at 9p21 in bladder cancer. *Hum Mol Genet* **4**: 1569–1577

Zhang W, Grasso L, McClain CD, Gambel AM, Cha Y, Travali S, Deisseroth AB and Merc E (1995) p53-independent induction of *WAF1/CIP1* in human leukemia cells is correlated with growth arrest accompanying monocyte/macrophage differentiation. *Cancer Res* **55**: 668–674

Zhang H, Chen ZH and Savarese TM (1996) Codeletion of the genes for p16INK4, methylthioadenosine phosphorylase, interferon-alpha1, interferon-beta1, and other 9p21 markers in human malignant cell lines. *Cancer Genet Cytogenet* **86**: 22–28

© Cancer Research Campaign 1998